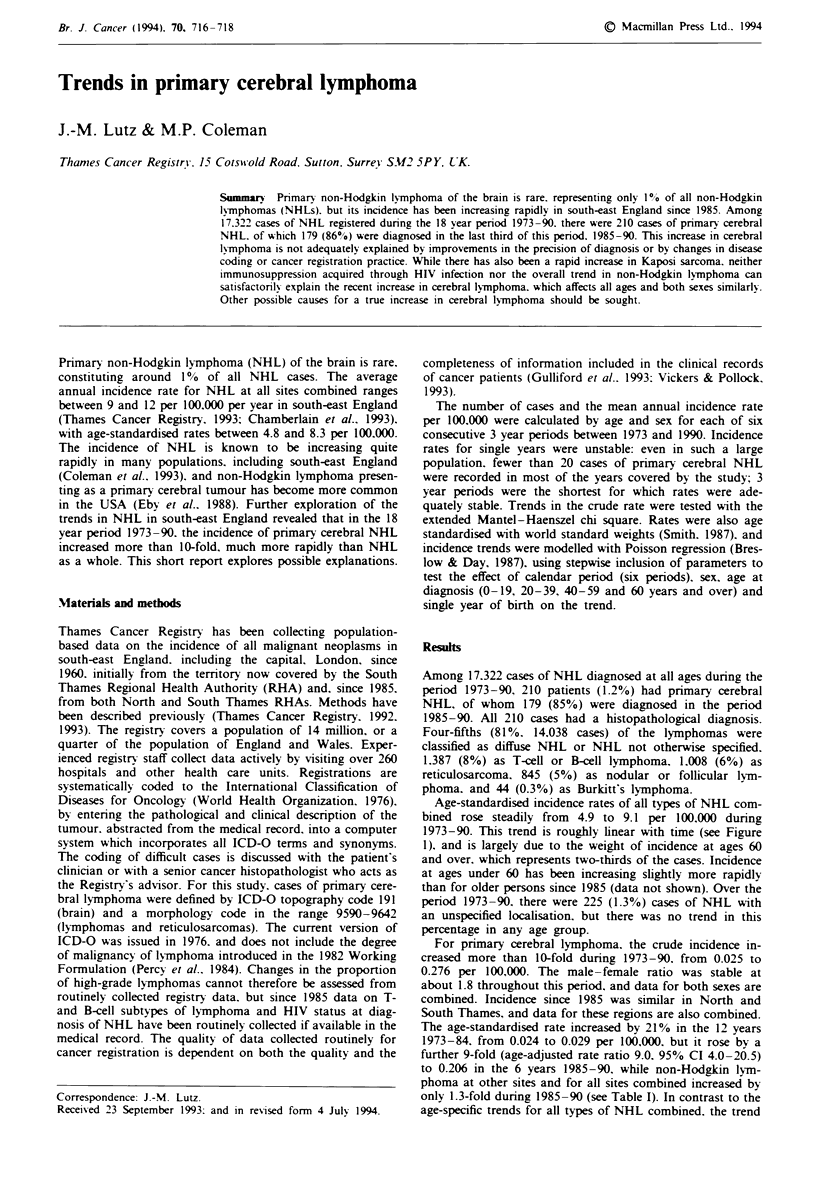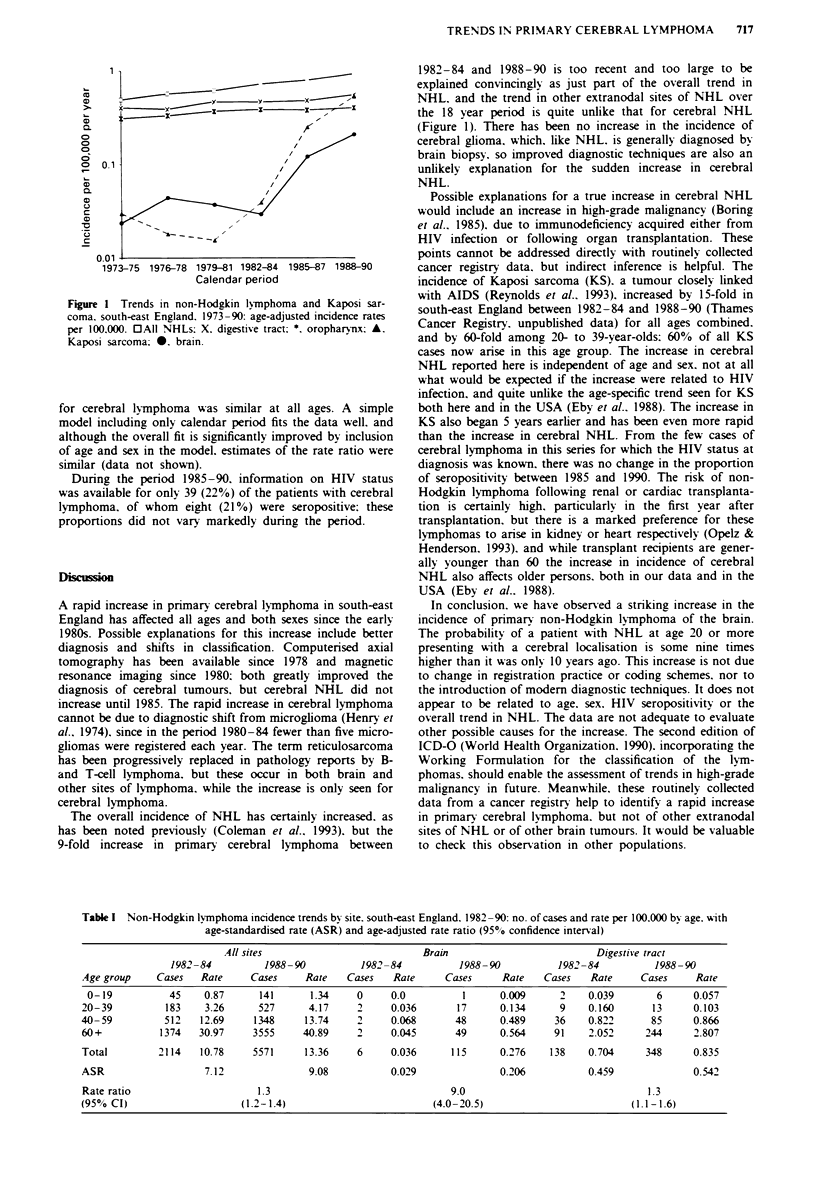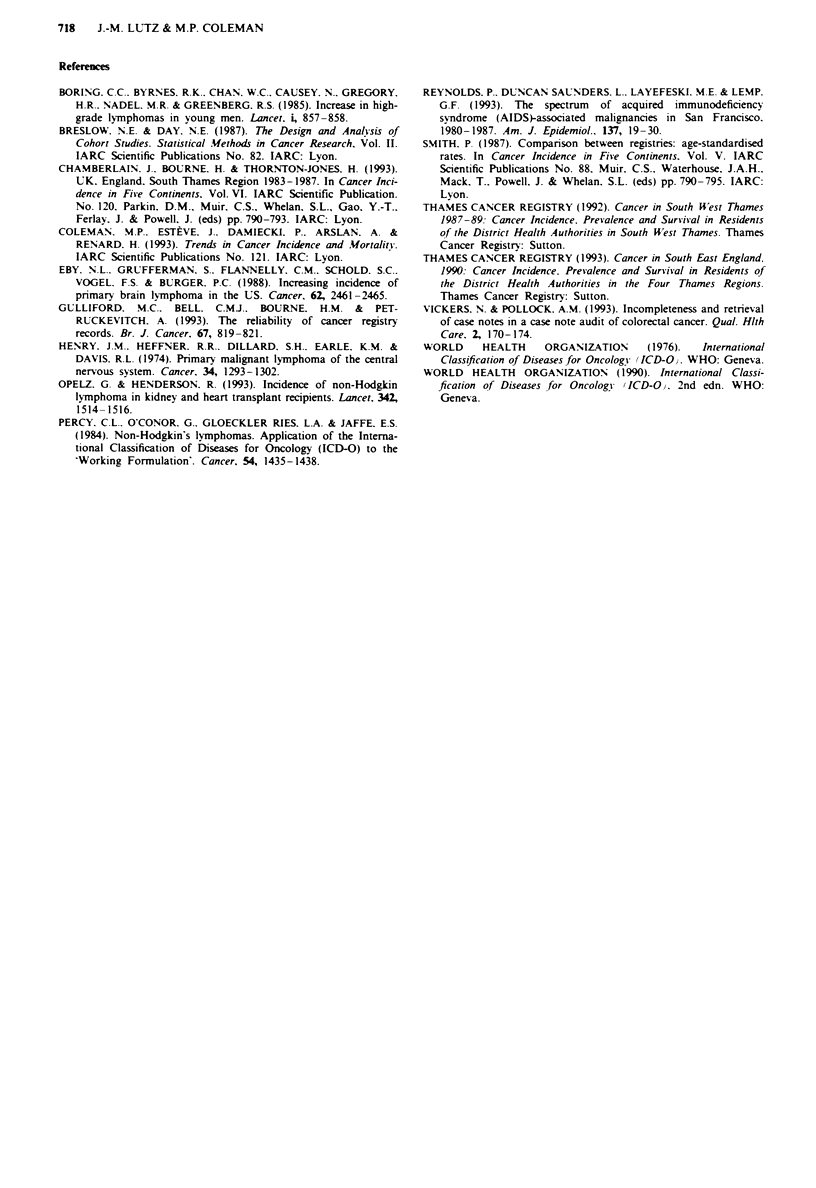# Trends in primary cerebral lymphoma.

**DOI:** 10.1038/bjc.1994.381

**Published:** 1994-10

**Authors:** J. M. Lutz, M. P. Coleman

**Affiliations:** Thames Cancer Registry, Sutton, Surrey, UK.

## Abstract

Primary non-Hodgkin lymphoma of the brain is rare, representing only 1% of all non-Hodgkin lymphomas (NHLs), but its incidence has been increasing rapidly in south-east England since 1985. Among 17,322 cases of NHL registered during the 18 year period 1973-90, there were 210 cases of primary cerebral NHL, of which 179 (86%) were diagnosed in the last third of this period, 1985-90. This increase in cerebral lymphoma is not adequately explained by improvements in the precision of diagnosis or by changes in disease coding or cancer registration practice. While there has also been a rapid increase in Kaposi sarcoma, neither immunosuppression acquired through HIV infection nor the overall trend in non-Hodgkin lymphoma can satisfactorily explain the recent increase in cerebral lymphoma, which affects all ages and both sexes similarly. Other possible causes for a true increase in cerebral lymphoma should be sought.


Br. J. Cancer (1994), 70, 716-718 © Macmillan Press Ltd., 1994

### Trends in primary cerebral lymphoma

J.-M. Lutz & M.P. Coleman

*Thames Cancer Registry, 15 Cotswold Road, Sutton, Surrey SM2 5PY, UK.*

**Summary** Primary non-Hodgkin lymphoma of the brain is rare, representing only 1% of all non-Hodgkin lymphomas (NHLs), but its incidence has been increasing rapidly in south-east England since 1985. Among 17,322 cases of NHL registered during the 18 year period 1973-90, there were 210 cases of primary cerebral NHL, of which 179 (86%) were diagnosed in the last third of this period, 1985-90. This increase in cerebral lymphoma is not adequately explained by improvements in the precision of diagnosis or by changes in disease coding or cancer registration practice. While there has also been a rapid increase in Kaposi sarcoma, neither immunosuppression acquired through HIV infection nor the overall trend in non-Hodgkin lymphoma can satisfactorily explain the recent increase in cerebral lymphoma, which affects all ages and both sexes similarly. Other possible causes for a true increase in cerebral lymphoma should be sought.

Primary non-Hodgkin lymphoma (NHL) of the brain is rare, constituting around 1% of all NHL cases. The average annual incidence rate for NHL at all sites combined ranges between 9 and 12 per 100,000 per year in south-east England (Thames Cancer Registry, 1993; Chamberlain *et al.*, 1993), with age-standardised rates between 4.8 and 8.3 per 100,000. The incidence of NHL is known to be increasing quite rapidly in many populations, including south-east England (Coleman *et al.*, 1993), and non-Hodgkin lymphoma presenting as a primary cerebral tumour has become more common in the USA (Eby *et al.*, 1988). Further exploration of the trends in NHL in south-east England revealed that in the 18 year period 1973-90, the incidence of primary cerebral NHL increased more than 10-fold, much more rapidly than NHL as a whole. This short report explores possible explanations.

### Materials and methods

Thames Cancer Registry has been collecting population-based data on the incidence of all malignant neoplasms in south-east England, including the capital, London, since 1960, initially from the territory now covered by the South Thames Regional Health Authority (RHA) and, since 1985, from both North and South Thames RHAs. Methods have been described previously (Thames Cancer Registry, 1992, 1993). The registry covers a population of 14 million, or a quarter of the population of England and Wales. Experienced registry staff collect data actively by visiting over 260 hospitals and other health care units. Registrations are systematically coded to the International Classification of Diseases for Oncology (World Health Organization, 1976), by entering the pathological and clinical description of the tumour, abstracted from the medical record, into a computer system which incorporates all ICD-O terms and synonyms. The coding of difficult cases is discussed with the patient's clinician or with a senior cancer histopathologist who acts as the Registry's advisor. For this study, cases of primary cerebral lymphoma were defined by ICD-O topography code 191 (brain) and a morphology code in the range 9590-9642 (lymphomas and reticulosarcomas). The current version of ICD-O was issued in 1976, and does not include the degree of malignancy of lymphoma introduced in the 1982 Working Formulation (Percy *et al.*, 1984). Changes in the proportion of high-grade lymphomas cannot therefore be assessed from routinely collected registry data, but since 1985 data on T- and B-cell subtypes of lymphoma and HIV status at diagnosis of NHL have been routinely collected if available in the medical record. The quality of data collected routinely for cancer registration is dependent on both the quality and the completeness of information included in the clinical records of cancer patients (Gulliford *et al.*, 1993; Vickers & Pollock, 1993).

The number of cases and the mean annual incidence rate per 100,000 were calculated by age and sex for each of six consecutive 3 year periods between 1973 and 1990. Incidence rates for single years were unstable: even in such a large population, fewer than 20 cases of primary cerebral NHL were recorded in most of the years covered by the study; 3 year periods were the shortest for which rates were adequately stable. Trends in the crude rate were tested with the extended Mantel-Haenszel chi square. Rates were also age standardised with world standard weights (Smith, 1987), and incidence trends were modelled with Poisson regression (Breslow & Day, 1987), using stepwise inclusion of parameters to test the effect of calendar period (six periods), sex, age at diagnosis (0-19, 20-39, 40-59 and 60 years and over) and single year of birth on the trend.

### Results

Among 17,322 cases of NHL diagnosed at all ages during the period 1973-90, 210 patients (1.2%) had primary cerebral NHL, of whom 179 (85%) were diagnosed in the period 1985-90. All 210 cases had a histopathological diagnosis. Four-fifths (81%, 14,038 cases) of the lymphomas were classified as diffuse NHL or NHL not otherwise specified, 1,387 (8%) as T-cell or B-cell lymphoma, 1,008 (6%) as reticulosarcoma, 845 (5%) as nodular or follicular lymphoma, and 44 (0.3%) as Burkitt's lymphoma.

Age-standardised incidence rates of all types of NHL combined rose steadily from 4.9 to 9.1 per 100,000 during 1973-90. This trend is roughly linear with time (see Figure 1), and is largely due to the weight of incidence at ages 60 and over, which represents two-thirds of the cases. Incidence at ages under 60 has been increasing slightly more rapidly than for older persons since 1985 (data not shown). Over the period 1973-90, there were 225 (1.3%) cases of NHL with an unspecified localisation, but there was no trend in this percentage in any age group.

For primary cerebral lymphoma, the crude incidence increased more than 10-fold during 1973-90, from 0.025 to 0.276 per 100,000. The male-female ratio was stable at about 1.8 throughout this period, and data for both sexes are combined. Incidence since 1985 was similar in North and South Thames, and data for these regions are also combined. The age-standardised rate increased by 21% in the 12 years 1973-84, from 0.024 to 0.029 per 100,000, but it rose by a further 9-fold (age-adjusted rate ratio 9.0, 95% CI 4.0-20.5) to 0.206 in the 6 years 1985-90, while non-Hodgkin lymphoma at other sites and for all sites combined increased by only 1.3-fold during 1985-90 (see Table I). In contrast to the age-specific trends for all types of NHL combined, the trend

Correspondence: J.-M. Lutz.
Received 23 September 1993; and in revised form 4 July 1994.

**Figure 1** Trends in non-Hodgkin lymphoma and Kaposi sarcoma, south-east England, 1973–90: age-adjusted incidence rates per 100,000. □All NHLs; X, digestive tract; *, oropharynx; ▲, Kaposi sarcoma; ●, brain.

for cerebral lymphoma was similar at all ages. A simple model including only calendar period fits the data well, and although the overall fit is significantly improved by inclusion of age and sex in the model, estimates of the rate ratio were similar (data not shown).

During the period 1985–90, information on HIV status was available for only 39 (22%) of the patients with cerebral lymphoma, of whom eight (21%) were seropositive; these proportions did not vary markedly during the period.

### Discussion

A rapid increase in primary cerebral lymphoma in south-east England has affected all ages and both sexes since the early 1980s. Possible explanations for this increase include better diagnosis and shifts in classification. Computerised axial tomography has been available since 1978 and magnetic resonance imaging since 1980; both greatly improved the diagnosis of cerebral tumours, but cerebral NHL did not increase until 1985. The rapid increase in cerebral lymphoma cannot be due to diagnostic shift from microglioma (Henry *et al.*, 1974), since in the period 1980–84 fewer than five microgliomas were registered each year. The term reticulosarcoma has been progressively replaced in pathology reports by B- and T-cell lymphoma, but these occur in both brain and other sites of lymphoma, while the increase is only seen for cerebral lymphoma.

The overall incidence of NHL has certainly increased, as has been noted previously (Coleman *et al.*, 1993), but the 9-fold increase in primary cerebral lymphoma between 1982–84 and 1988–90 is too recent and too large to be explained convincingly as just part of the overall trend in NHL, and the trend in other extranodal sites of NHL over the 18 year period is quite unlike that for cerebral NHL (Figure 1). There has been no increase in the incidence of cerebral glioma, which, like NHL, is generally diagnosed by brain biopsy, so improved diagnostic techniques are also an unlikely explanation for the sudden increase in cerebral NHL.

Possible explanations for a true increase in cerebral NHL would include an increase in high-grade malignancy (Boring *et al.*, 1985), due to immunodeficiency acquired either from HIV infection or following organ transplantation. These points cannot be addressed directly with routinely collected cancer registry data, but indirect inference is helpful. The incidence of Kaposi sarcoma (KS), a tumour closely linked with AIDS (Reynolds *et al.*, 1993), increased by 15-fold in south-east England between 1982–84 and 1988–90 (Thames Cancer Registry, unpublished data) for all ages combined, and by 60-fold among 20- to 39-year-olds; 60% of all KS cases now arise in this age group. The increase in cerebral NHL reported here is independent of age and sex, not at all what would be expected if the increase were related to HIV infection, and quite unlike the age-specific trend seen for KS both here and in the USA (Eby *et al.*, 1988). The increase in KS also began 5 years earlier and has been even more rapid than the increase in cerebral NHL. From the few cases of cerebral lymphoma in this series for which the HIV status at diagnosis was known, there was no change in the proportion of seropositivity between 1985 and 1990. The risk of non-Hodgkin lymphoma following renal or cardiac transplantation is certainly high, particularly in the first year after transplantation, but there is a marked preference for these lymphomas to arise in kidney or heart respectively (Opelz & Henderson, 1993), and while transplant recipients are generally younger than 60 the increase in incidence of cerebral NHL also affects older persons, both in our data and in the USA (Eby *et al.*, 1988).

In conclusion, we have observed a striking increase in the incidence of primary non-Hodgkin lymphoma of the brain. The probability of a patient with NHL at age 20 or more presenting with a cerebral localisation is some nine times higher than it was only 10 years ago. This increase is not due to change in registration practice or coding schemes, nor to the introduction of modern diagnostic techniques. It does not appear to be related to age, sex, HIV seropositivity or the overall trend in NHL. The data are not adequate to evaluate other possible causes for the increase. The second edition of ICD-O (World Health Organization, 1990), incorporating the Working Formulation for the classification of the lymphomas, should enable the assessment of trends in high-grade malignancy in future. Meanwhile, these routinely collected data from a cancer registry help to identify a rapid increase in primary cerebral lymphoma, but not of other extranodal sites of NHL or of other brain tumours. It would be valuable to check this observation in other populations.

**Table I** Non-Hodgkin lymphoma incidence trends by site, south-east England, 1982–90: no. of cases and rate per 100,000 by age, with age-standardised rate (ASR) and age-adjusted rate ratio (95% confidence interval)

| | *All sites* | | | | *Brain* | | | | *Digestive tract* | | | |
|---|---|---|---|---|---|---|---|---|---|---|---|---|
| | *1982–84* | | *1988–90* | | *1982–84* | | *1988–90* | | *1982–84* | | *1988–90* | |
| *Age group* | *Cases* | *Rate* | *Cases* | *Rate* | *Cases* | *Rate* | *Cases* | *Rate* | *Cases* | *Rate* | *Cases* | *Rate* |
| 0–19 | 45 | 0.87 | 141 | 1.34 | 0 | 0.0 | 1 | 0.009 | 2 | 0.039 | 6 | 0.057 |
| 20–39 | 183 | 3.26 | 527 | 4.17 | 2 | 0.036 | 17 | 0.134 | 9 | 0.160 | 13 | 0.103 |
| 40–59 | 512 | 12.69 | 1348 | 13.74 | 2 | 0.068 | 48 | 0.489 | 36 | 0.822 | 85 | 0.866 |
| 60+ | 1374 | 30.97 | 3555 | 40.89 | 2 | 0.045 | 49 | 0.564 | 91 | 2.052 | 244 | 2.807 |
| Total | 2114 | 10.78 | 5571 | 13.36 | 6 | 0.036 | 115 | 0.276 | 138 | 0.704 | 348 | 0.835 |
| ASR | | 7.12 | | 9.08 | | 0.029 | | 0.206 | | 0.459 | | 0.542 |
| Rate ratio (95% CI) | | | 1.3 (1.2–1.4) | | | | 9.0 (4.0–20.5) | | | | 1.3 (1.1–1.6) | |

### References

BORING, C.C., BYRNES, R.K., CHAN, W.C., CAUSEY, N., GREGORY, H.R., NADEL, M.R. & GREENBERG, R.S. (1985). Increase in high-grade lymphomas in young men. *Lancet*, **i**, 857-858.

BRESLOW, N.E. & DAY, N.E. (1987). *The Design and Analysis of Cohort Studies. Statistical Methods in Cancer Research*, Vol. II. IARC Scientific Publications No. 82. IARC: Lyon.

CHAMBERLAIN, J., BOURNE, H. & THORNTON-JONES, H. (1993). UK, England, South Thames Region 1983-1987. In *Cancer Incidence in Five Continents*, Vol. VI. IARC Scientific Publication. No. 120. Parkin, D.M., Muir, C.S., Whelan, S.L., Gao, Y.-T., Ferlay, J. & Powell, J. (eds) pp. 790-793. IARC: Lyon.

COLEMAN, M.P., ESTÈVE, J., DAMIECKI, P., ARSLAN, A. & RENARD, H. (1993). *Trends in Cancer Incidence and Mortality*. IARC Scientific Publications No. 121. IARC: Lyon.

EBY, N.L., GRUFFERMAN, S., FLANNELLY, C.M., SCHOLD, S.C., VOGEL, F.S. & BURGER, P.C. (1988). Increasing incidence of primary brain lymphoma in the US. *Cancer*, **62**, 2461-2465.

GULLIFORD, M.C., BELL, C.M.J., BOURNE, H.M. & PETRUCKEVITCH, A. (1993). The reliability of cancer registry records. *Br. J. Cancer*, **67**, 819-821.

HENRY, J.M., HEFFNER, R.R., DILLARD, S.H., EARLE, K.M. & DAVIS, R.L. (1974). Primary malignant lymphoma of the central nervous system. *Cancer*, **34**, 1293-1302.

OPELZ, G. & HENDERSON, R. (1993). Incidence of non-Hodgkin lymphoma in kidney and heart transplant recipients. *Lancet*, **342**, 1514-1516.

PERCY, C.L., O'CONOR, G., GLOECKLER RIES, L.A. & JAFFE, E.S. (1984). Non-Hodgkin's lymphomas. Application of the International Classification of Diseases for Oncology (ICD-O) to the 'Working Formulation'. *Cancer*, **54**, 1435-1438.

REYNOLDS, P., DUNCAN SAUNDERS, L., LAYEFESKI, M.E. & LEMP, G.F. (1993). The spectrum of acquired immunodeficiency syndrome (AIDS)-associated malignancies in San Francisco, 1980-1987. *Am. J. Epidemiol.*, **137**, 19-30.

SMITH, P. (1987). Comparison between registries: age-standardised rates. In *Cancer Incidence in Five Continents*, Vol. V. IARC Scientific Publications No. 88, Muir, C.S., Waterhouse, J.A.H., Mack, T., Powell, J. & Whelan, S.L. (eds) pp. 790-795. IARC: Lyon.

THAMES CANCER REGISTRY (1992). *Cancer in South West Thames 1987-89: Cancer Incidence, Prevalence and Survival in Residents of the District Health Authorities in South West Thames*. Thames Cancer Registry: Sutton.

THAMES CANCER REGISTRY (1993). *Cancer in South East England, 1990: Cancer Incidence, Prevalence and Survival in Residents of the District Health Authorities in the Four Thames Regions*. Thames Cancer Registry: Sutton.

VICKERS, N. & POLLOCK, A.M. (1993). Incompleteness and retrieval of case notes in a case note audit of colorectal cancer. *Qual. Hlth Care*, **2**, 170-174.

WORLD HEALTH ORGANIZATION (1976). *International Classification of Diseases for Oncology (ICD-O)*. WHO: Geneva.

WORLD HEALTH ORGANIZATION (1990). *International Classification of Diseases for Oncology (ICD-O)*, 2nd edn. WHO: Geneva.